# Distribution, conservation status and proposed measures for preservation of *Radiodiscus* microgastropods in Chile

**DOI:** 10.7717/peerj.14027

**Published:** 2023-01-10

**Authors:** Gonzalo A. Collado, Rodrigo B. Salvador, Marcela Vidal, Fernanda Parra Aravena, Vannia Delgado, Cristian Torres-Díaz

**Affiliations:** 1 Departamento de Ciencias Básicas, Universidad del Bío-Bío, Chillán, Ñuble, Chile; 2 Grupo de Investigación en Biodiversidad and Cambio Global, Universidad del Bío-Bío, Chillán, Chile; 3 Museum of New Zealand Te Papa Tongarewa, Wellington, New Zealand; 4 Department of Arctic and Marine Biology, Faculty of Biosciences, Fisheries and Economics, UiT—The Arctic University of Norway, Tromsø, Norway; 5 The Arctic University Museum of Norway, UiT—The Arctic University of Norway, Tromsø, Norway

**Keywords:** Conservation biology, Endangered species, IUCN Red List, Land snails, Micromollusks, NatureServe

## Abstract

The genus *Radiodiscus* includes minute terrestrial snails occurring throughout the American continent. We assessed the conservation status of eight poorly known Chilean *Radiodiscus* species using the International Union for Conservation of Nature (IUCN) and NatureServe categories and criteria. Under the IUCN guidelines the species were assessed using the Criterion B of geographic range, which considers the extent of occurrence (EOO) and area of occupancy (AOO) as subcriteria. For NatureServe we used these two parameters plus the number of occurrences, ecological viability, and threats. Considering species rarity and possible sampling bias, we also used ecological niche modeling to determine climate and environmental tolerances and predict potential species distributions analyzing bioclimatic and geographical layers. *Radiodiscus australis*, *R. coarctatus* and *R. quillajicola* were listed as Critically Endangered by IUCN and NatureServe standards; *R. coppingeri*, *R. flammulatus*, *R. magellanicus* and *R. villarricensis* as Endangered by both methods; while *R. riochicoensis* as Endangered by IUCN standards and Vulnerable by NatureServe standards. Niche modeling results indicated that *Radiodiscus* species respond to different environmental conditions and that the predicted distribution areas contain suitable habitats beyond the current ranges, which may be helpful for future management plans. Nature-based sport tourism, forestry activities, urbanization, roads, pollution, mining, forest fires, livestock, volcanism, tsunamis, soil erosion and introduced species are among the major threats affecting these snails. Based on the low number of occurrences and the threats identified, the most at-risk species are *R. coarctatus* and *R. quillajicola* (one record), *R. australis* (two records) and *R. villarricensis* (three records); the latter two lacking occurrences within protected areas. Compiling our findings, we propose a list of actions to preserve Chilean *Radiodiscus* species.

## Introduction

Habitat loss is considered the major threat to biodiversity (Rawat & Agarwal, 2015). However, other human-induced factors, such as the introduction of exotic species, environmental pollution and rapid climate change, can also lead species to extinction (Richards, 2002; Matthies et al., 2004; Thomas et al., 2004; Mason, 2015). The Mediterranean climate zone of central Chile harbors two of the 25 global biodiversity hotspots (Myers et al., 2000). However, like other important ecosystems around the world, these have been drastically affected by human activities (*e.g.*, habitat loss, urbanization, poaching, overgrazing, pollution, mining, energy generation, roads, fires, introduced species), which impacts the local biota and the ecosystem services they provide (CONAMA, 2003; Soto et al., 2006; Valderrama, Contreras-Reyes & Carrasco, 2018; Carranza et al., 2020; Moat et al., 2021). According to the MMA (2021), over 33,000 native species have been described from Chile, of which around 22–25% are endemic. Thus, the totality of Chilean biota account for about 1.5% of the species described for the world (MMA, 2021). This relatively high biodiversity has been attributed to the country’s biogeographical isolation due to the barriers surrounding the territory: the Andes to the east, the Pacific Ocean to the west, the Atacama Desert to the north, and the Antarctic Ocean to the south.

The genus *Radiodiscus* Pilsbry in Pilsbry & Ferriss, 1906 (Charopidae) comprises around 25 species of minute terrestrial snails distributed throughout the Americas from western USA to Tierra del Fuego (Smith, 1881; Hylton Scott, 1970; Schileyko, 2001; Rutherford, 2020). The taxonomic treatment of these micromollusks has been historically difficult due to their small size, since at an adult age, the individuals are generally less than 2 mm in shell length (Miquel & Bellosi, 2007; Rutherford, 2020). Furthermore, they live in the soil and leaf litter and are thus typically overlooked in collection efforts and underrepresented in natural history collections (Salvador et al., 2020). According to molecular phylogenetic studies, Charopidae is paraphyletic and so is the genus *Radiodiscus*: specimens of *Radiodiscus* from Chile and Brazil, for instance, form unrelated branches in the family’s phylogenetic tree (Salvador et al., 2020). The placement of the type species of the genus, *Radiodiscus millecostatus* Pilsbry & Ferriss, 1906 (type locality in Arizona, USA; Pilsbry & Ferriss, 1906) in the tree remains unknown. In the few checklists that include Chilean *Radiodiscus* species, they were historically classified in the family Endodontidae (Stuardo & Vega, 1985; Valdovinos, 1999), but are currently placed in the family Charopidae following more recent taxonomic treatments based on morphological and molecular data (Aldea, Novoa & Rosenfeld, 2019; Schileyko, 2001; Salvador, 2021). As currently understood, *Radiodiscus* is potentially a wastebasket taxon that houses species whose protoconch sculpture pattern consists in numerous spiral cordlets (Zilch, 1959; Schileyko, 2001; Salvador, 2021). Despite these uncertainties, this genus remains a useful taxonomic umbrella for conservation purposes. In Chile, the degree of threat to which *Radiodiscus* species are exposed is not known.

A high degree of endemism has developed in the Chilean territory, with many species having one or few populations. Concordantly, few records may be a consequence of a restricted habitat, not necessarily sampling bias. Hortal et al. (2015) summarized the seven main shortfalls that affect the knowledge of biodiversity in all taxonomic groups, covering taxonomy, distribution, abundance, phylogenetic relationships, adaptations, ecological niche and interactions, and *Radiodiscus* does not escape this situation. Although the Chilean species of this genus are poorly known in many aspects, the lack of “perfect” data should not impair conservation assessments (Pokharel, Ludwig & Storch, 2016). Thus, these limitations offer an opportunity to evaluate the conservation status of Chilean *Radiodiscus* species, appraise the threats they face, their known and potential geographic distribution, as well as the environmental and climatic variables influencing their habitats. A necessary step to prevent extinction of a species is making opportune assessments of the conservation status (Bernardos, Amado & Amich, 2006). The International Union for Conservation of Nature (IUCN) Red List of Threatened Species (IUCN, 2001) and the Natural Heritage Network Element Ranking System (NatureServe, 2006) are two international initiatives created for this purpose, and most countries have similar national-scale organizations. In Chile, the institution in charge of evaluating the conservation status of species is the Ministry of the Environment (MMA, 2021), based on the IUCN’s guidelines (Squeo et al., 2010). On the other hand, environmental niche modeling, ecological niche modeling or species distribution modeling has achieved significant development in the last two decades, covering, in addition to the current and potential distribution of native and invasive species, aspects regarding climatic change, conservation biology, eco-restoration sites and eco-cultural reconstruction, species delimitation, estimate ancestral distribution, investigate speciation processes, discoveries of new species, subspecies, cryptic species and areas of endemism (Peterson & Holt, 2003; Graham et al., 2004; Wiens & Graham, 2005; Banks et al., 2006; Raxworthy et al., 2007; Hammerson et al., 2017; Carvajal-Hernández et al., 2020).

The aims of the present study were to (1) estimate the current distribution of the Chilean *Radiodiscus* species (*sensu lato*) and assess their conservation status according to categories and criteria implemented by the IUCN (2001, 2014, 2019) and NatureServe (2006) and (2) determine climate and environmental variables influencing the habitat of species and predict their potential distributions using ecological niche modeling. Finally, we propose measures to conserve the species based on occurrence records, geographic distribution, conservation status, habitat availability and threats.

## Materials & Methods

### Chilean *Radiodiscus* species

We follow the currently accepted literature in assigning eight Chilean species to the genus *Radiodiscus* (Stuardo & Vega, 1985; Valdovinos, 1999; Aldea, Novoa & Rosenfeld, 2019). Three of those species are endemic to Chile, namely *Radiodiscus coarctatus* Hylton Scott, 1979, *Radiodiscus quillajicola* Vargas-Almonacid, 2000 and *Radiodiscus villarricensis* Miquel & Barker, 2009, while the remaining five species occur in Chile and Argentina: *Radiodiscus australis* Hylton Scott, 1970, *Radiodiscus coppingeri* (Smith, 1881), *Radiodiscus flammulatus* Hylton Scott, 1975, *Radiodiscus magellanicus* (Smith, 1881) and *Radiodiscus riochicoensis* Crawford, 1939.

Information on the distribution and biology of these species was likewise obtained from the literature, through different sources including original descriptions, subsequent taxonomic contributions, distribution records and species checklists (Smith, 1881; Pilsbry, 1911; Crawford, 1939; Hylton Scott, 1957; Hylton Scott, 1963; Hylton Scott, 1970; Hylton Scott, 1975; Hylton Scott, 1979; Stuardo & Vega, 1985; Fonseca & Thomé, 1993; Fonseca & Thomé, 1994; Valdovinos, 1999; Vargas-Almonacid, 2000; Sielfeld, 2001; Miquel & Bellosi, 2007; Tablado & Mantinian, 2004; Miquel & Bellosi, 2007; Miquel & Barker, 2009; Miquel & Aguirre, 2011; Aldea, Novoa & Rosenfeld, 2019; Salvador et al., 2021). In addition, we also include GBIF records provided by prominent malacologists or curators of mollusks collections (Darrigran & Damborenea, 2017; Tablado & Rodríguez, 2021; Goud, van der Bijl & Creuwels, 2022), as well as Hylton Scott (1970) and Hylton Scott (1979) (see Table S1 for links to these sources).

Six further species of micromollusks originally described by Philippi (1855) under the genus *Helix* Linnaeus, 1758 are considered of uncertain affinities (Stuardo & Vega, 1985) but have been recently listed as species of *Radiodiscus* in platforms such as GBIF (2021) and MolluscaBase (2021) without any explanation for the decision. Therefore, those six “*Helix*” species were not included in the present study.

### Conservation status

Historical localities recovered from the literature for each species of *Radiodiscus* are available in Table S1. Those localities lacking geographic coordinates were georeferenced *via* the radio-point method. This procedure consists in establishing a central point within the area reported in the respective publication and to define a circumference around it that includes the most probable collection site and its associated uncertainty (Wieczorek, Guo & Hijmans, 2004; Escobar et al., 2016). Geographic coordinates and radius of uncertainty were obtained using the software Google Earth (v.7.3.3.7786; Google Inc., Mountain View, CA, USA).

The IUCN categorizes species according to five criteria: Criterion A refers to population size reduction, Criterion B to geographic range, Criterion C to small population size and decline, Criterion D to very small or restricted population, and Criterion E to quantitative analysis of the probability of extinction. We assessed the conservation status of the Chilean *Radiodiscus* species using Criterion B because no information is available for the application of criteria A, C, and E in this group. Criterion B considers geographic range of species, involving subcriterion B1, based on the extent of occurrence (EOO), and subcriterion B2, based on the area of occupancy (AOO). Basically, the EOO is the area covered by the polygon formed by a union line that encloses all occurrences of a species while the AOO is the sum of the estimated areas around each occurrence. In the latter case, a 2 km^2^ grid cell was used (Bland et al., 2016; IUCN, 2019). To calculate the parameters EOO and AOO and to automatically obtain a preliminary conservation category, the geographic coordinates of each species were introduced into the GeoCAT program (Bachman et al., 2011). We also apply sub-criterion D2 (Criterion D), for species with few locations (≤ 5), an AOO ¡ 20 km^2^ and at least one plausible threat. If a species meets these conditions it must be listed as Vulnerable: D2. The conservation categories that can be obtained using this tool are Least Concern (LC), Near Threatened (NT), Vulnerable (VU), Endangered (EN) and Critically Endangered (CR).

To assess the conservation status based on NatureServe’s standards (Faber-Langendoen et al., 2012; Master et al., 2012) we used the Conservation Rank Calculator tool (NatureServe, 2021), which allows entering the data of the species to obtain a conservation category based on the range extent (equivalent to EOO), area of occupancy (AOO), numbers of occurrences, ecological viability, population trends and threats (Table 1). NatureServe also allows the researcher to specify the geographic level of the conservation assessment: Global (G), National (N), and Subnational (S) (Faber-Langendoen et al., 2012; Master et al., 2012). Under this methodology, the species may be assigned to one of the five categories: Critically Imperiled (G1/N1/S1), Imperiled (G2/N2/S2), Vulnerable (G3/N3/S3), Apparently Secure (G4/N4/S4) and Secure (G5/N5/S5). We determined ecological viability in the sense of habitat quality using satellite images on Google Earth exploring whether the occurrence records and surrounding areas of *Radiodiscus* species still have natural coverage thus providing a suitable environment to live (“Good” habitat in Table S1). The threats facing species were obtained merging species occurrence records with road, hydrographic and urban development vector layers available in the Library of the National Congress of Chile (https://www.bcn.cl/siit/mapas_vectoriales), which were analyzed in QGIS v.3.22.7 software (QGIS Development Team, 2021), as well as information obtained from the literature, government organizations and the media. Operationally, in the Conservation Rank Calculator we first enter the threats identified previously in each species assigning the highest level of threat in the “Threats Assessment Worksheet” to then record in the “Calculator Form Worksheet” the data available *per* taxon regarding EOO, AOO, occurrence records, ecological viability and threats. The present study was carried out at the national level (N), without considering population trends due to lack of information.

**Table 1:** Data used for conservation status assessment of Chilean species of *Radiodiscus* based on NatureServe standards.

**Species**	**O**	**EOO (km^2^)**	**AOO (km^2^)**	**Occurrences with good ecological viability**	**Threat impact**
*Radiodiscus australis*	1–5	<100	6–25	1–3	Very high
*Radiodiscus coarctatus*	1–5	<100	3–5	1–3	Very high
*Radiodiscus coppingeri*	21–80	200,000–2,500,000	126–500	13–40	Very high
*Radiodiscus flammulatus*	6–20	20,000–200,000	26–125	4–12	High
*Radiodiscus magellanicus*	6–20	200,000–2,500,000	26–125	13–40	Very high
*Radiodiscus quillajicola*	1–5	<100	3–5	1–3	High
*Radiodiscus riochicoensis*	6–20	200,000–2,500,000	26–125	13–40	High
*Radiodiscus villarricensis*	1–5	1,000–5,000	6–25	1–3	High

**Notes.**

Abbreviatures O occurrences EOO extent of occurrence AOO area of occupancy

### Mapping of occurrences over protected areas

The Chilean National System of Protected Areas (SNASPE, “Sistema Nacional de Áreas Silvestres Protegidas del Estado”) has established 118 protected areas to preserve the country’s natural heritage (Root-Bernstein et al., 2013; Subsecretaría de Turismo, 2015). These areas include National Parks, National Reserves, Biosphere Reserves, Natural Monuments, Ramsar Sites, National Protected Goods, Marine Reserves and Coastal Marine Protected Areas. The total coverage of those areas is about 20% of the national territory (Root-Bernstein et al., 2013; Subsecretaría de Turismo, 2015). To determine if any of the species occurs within any of the protected areas, we built maps in QGIS previously entering vector layers of the country’s regions and protected areas available at the Library of National Congress of Chile (https://www.bcn.cl/siit/mapas_vectoriales). At the same time, the EOO, AOO and occurrence records of the species were downloaded from GeoCAT in KML format to be used in QGIS.

### Ecological niche modeling

A total of 19 bioclimatic variables (Fick & Hijmans, 2017) were downloaded from the WorldClim database version 2.0 (http://www.worldclim.org) and used in conjunction with environmental variables obtained from the same platform (solar radiation, evaporation, elevation, wind) [resolution of ∼1 km^2^(30 s)]. Each variable was merged with a vector geographic layer of the Chilean territory available in the Library of the National Congress of Chile (https://www.bcn.cl/siit/mapas_vectoriales) in a new raster variable (.ASCII) using ArcGIS v.10.8 software (Esri, Redlands, CA, USA). To estimate potential species distribution, we uploaded species occurrence records and the complete set of variables in the Maxent v.3.4.4 software (Phillips, Dudík & Schapire, 2004) to run four models *per* species computed using the *logistic* option. Model 1 was performed including all climatic and environmental variables and default settings. Model 2 included all variables with a setting of 25% of the occurrences for model testing. Model 3 included only the variables that most contributed to the model and that were obtained in Model 2, with default settings. Model 4 was the Model 3 but setting 25% for model testing. The performance of the model was validated with the AUC (area under the curve) based on the Receiver Operating Characteristic (ROC), which estimates values from 0.5 to 1.0, with values above 0.8 depicting a good fit of the model (Loo, Mac Nally & Lake, 2007). The importance of the variables that best explained the presence of *Radiodiscus* species was evaluated with the Jackknife test, which identifies the variable with highest gain regarding the other variables. The potential species distribution map resulting from the selected model was converted from ASCII to raster in ArcGIS for visualization.

## Results

**Figure 1:**
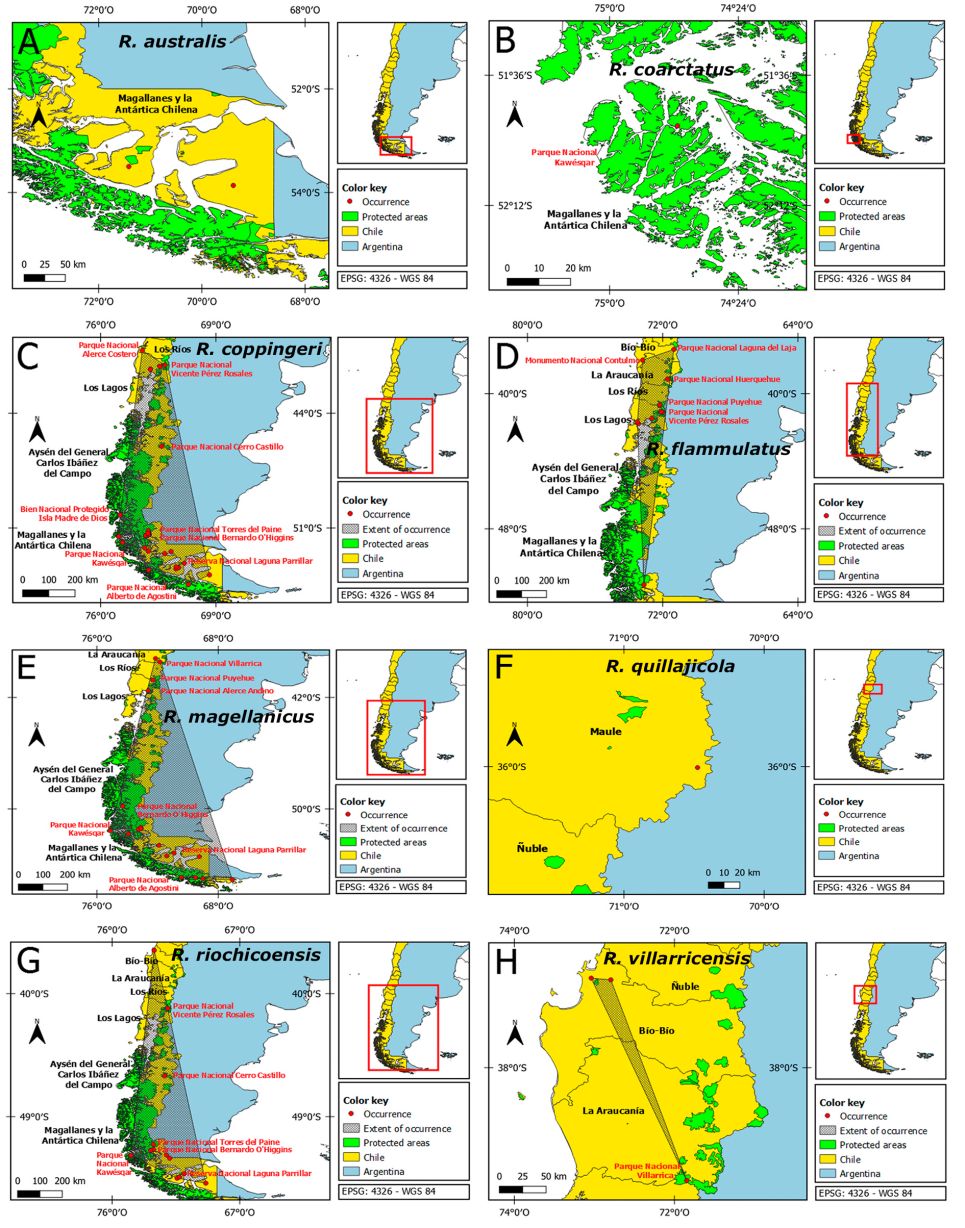
Mapping of occurrences *Radiodiscus* spp. in Chile, highlighting protected areas. (A) *Radiodiscus australis*, (B) *Radiodiscus coarctatus*, (C) *Radiodiscus coppingeri*, (D) *Radiodiscus flammulatus*, (E) *Radiodiscus magellanicus*, (F) *Radiodiscus quillajicola*, (G) *Radiodiscus riochicoensis*, (H) *Radiodiscus villaricensis*. The maps were created using GeoCAT (http://geocat.kew.org/) and QGIS (http://www.arcgis.com/index.html) software. (Maps: F Parra, V Delgado and GA Collado).

Considering historical records, the genus *Radiodiscus* is distributed in Chile from the country’s central-south zone to the austral zone (Figs. 1A–1H; Table S1). The areas of occupancy still have natural coverage that can support species, even for those populations whose records in the literature are decades old (Table S1). The species with the largest geographic distributions are *R. flammulatus* and *R. riochicoensis*, which range from the Bío-Bío Region to the Magallanes y Antártica Chilena Region. *Radiodiscus quillajicola* is the northernmost species of the genus in Chile. Four species have less than four occurrences at national level and four others have between 10 and 34 occurrences, and only *R. coppingeri* has over 20 occurrences (Table 2). The species restricted to a single locality are *R. coarctatus* (Magallanes y Antártica Chilena Region in southern Patagonia) and *R. quillajicola* (Maule Region in the central area of the country). Six species have at least one occurrence within protected areas, namely: *R. coarctatus*, *R. coppingeri*, *R. flammulatus*, *R. magellanicus*, *R. riochicoensis* and *R. villarricensis* (Table 2).

**Table 2:** Distributional data and conservation status based on IUCN Red List and NatureServe categories and criteria for Chilean species of *Radiodiscus*.

**Species**	**O**	**OPA**	**EOO (km^2^)**	**AOO (km^2^)**	**IUCN**			**NS**
					**EOO**	**AOO**	**D**	
*R. australis*	2	0	0.005^*^	8	CR B1ab(iii)	CR B2ab(iii)	D2	N1
*R. coarctatus*	1	1	0.002^*^	4	CR B1ab(iii)	CR B2ab(iii)	D2	N1
*R. coppingeri*	34	21	361,932	128	LC	EN B2ab(iii)		N2
*R. flammulatus*	10	6	141,827	40	LC	EN B2ab(iii)		N2
*R. magellanicus*	19	13	461,888	76	LC	EN B2ab(iii)		N2
*R. quillajicola*	1	0	0.003^*^	4	CR B1ab(iii)	CR B2ab(iii)	D2	N1
*R. riochicoensis*	15	9	335,700	56	LC	EN B2ab(iii)		N3
*R. villarricensis*	3	1	3,061	12	EN B2ab(iii)	EN B2ab(iii)	D2	N2

**Notes.**

Threatened IUCN categories CR Critically Endangered EN Endangered LC Least Concern D2 Vulnerable

Threatened NatureServe categories N1 Critically Imperiled N2 Imperiled N3 Vulnerable

Other abbreviatures NS NatureServe O occurrences OPA occurrences in protected areas EOO extent of occurrence AOO area of occupancy

* Values of EOO should be changed to make them equal to AOO (IUCN, 2019).

Based on IUCN standards, GeoCAT outputs showed EOO values ranging between 0.002 km^2^ (*R. coarctatus*) and 461,888 km^2^ (*R. magellanicus*), and AOO values between 4 km^2^ (*R. coarctatus* and *R. quillajicola*) and 128 km^2^ (*R. coppingeri*). Since EOO is greater than AOO, empirical values of EOO lower than AOO should be changed to make them equal to AOO (IUCN, 2014; IUCN, 2019). According to EOO, three species were classified as Critically Endangered (CR), four as Least Concern (LC) and one Endangered (EN); conversely, by using AOO, three species were classified as CR and five as EN (Table 2). *Radiodiscus australis* is listed as Critically Endangered CR B1ab(iii)+2ab(iii) since the EOO ¡ 100 km^2^ (B1), AOO ¡ 10 km^2^ (B2), number of locations =2 (a) and decline in habitat quality due to human activities and invasive species [b(iii)]. *Radiodiscus coarctatus* and *R. quillajicola* are listed as CR B1ab(iii)+2ab(iii) since the EOO ¡ 100 km^2^ (B1), AOO ¡ 10 km^2^ (B2), number of locations = 1 (a) and decline in habitat quality due to human activities and natural disasters [b(iii)]. *Radiodiscus villarricensis* is listed as EN B1ab(iii)+2ab(iii) since the EOO ¡ 5000 km^2^ (B1), AOO ¡ 500 km^2^ (B2), number of locations =3 (a) and decline in habitat quality due to human activities and natural disasters [b(iii)]. *Radiodiscus coppingeri*, *R. flammulatus*, *R. magellanicus* and *R. riochicoensis* are listed as EN B2ab(iii) since the AOO ¡ 500 km^2^ (B2), AOO severely fragmented assumed from the great distance between the known populations and low vagility of species (a), and decline in habitat quality due to human activities and natural disasters [b(iii)]. Using the D2 subcriterion, four species were also listed as Vulnerable (D2) (Table 2). Based on NatureServe standards, the threats impact obtained using the Conservation Rank Calculator was “High” for four species and “Very high” for four others (Table 1). Under this system, three species were classified as Critically Imperiled (N1), four as Imperiled (N2) and one as Vulnerable (N3) (Table 2).

We did not find reports in the scientific literature of any specific threats affecting Chilean *Radiodiscus* species. However, we detected at least 17 potential threats with the other methods proposed, which are applicable to most terrestrial snails in the country: nature and sports tourism, forestry activities, illegal logging of native forests, livestock, heavy rainfall events, introduced species, activities associated with artisanal fishing, soil erosion, pollution, forest fires, urbanization, volcanism, tsunamis, mining, highways and roads, hydroelectric plants, and droughts (Table S2). The most common threats affecting all species are tourism and geological events (volcanism).

**Figure 2:**
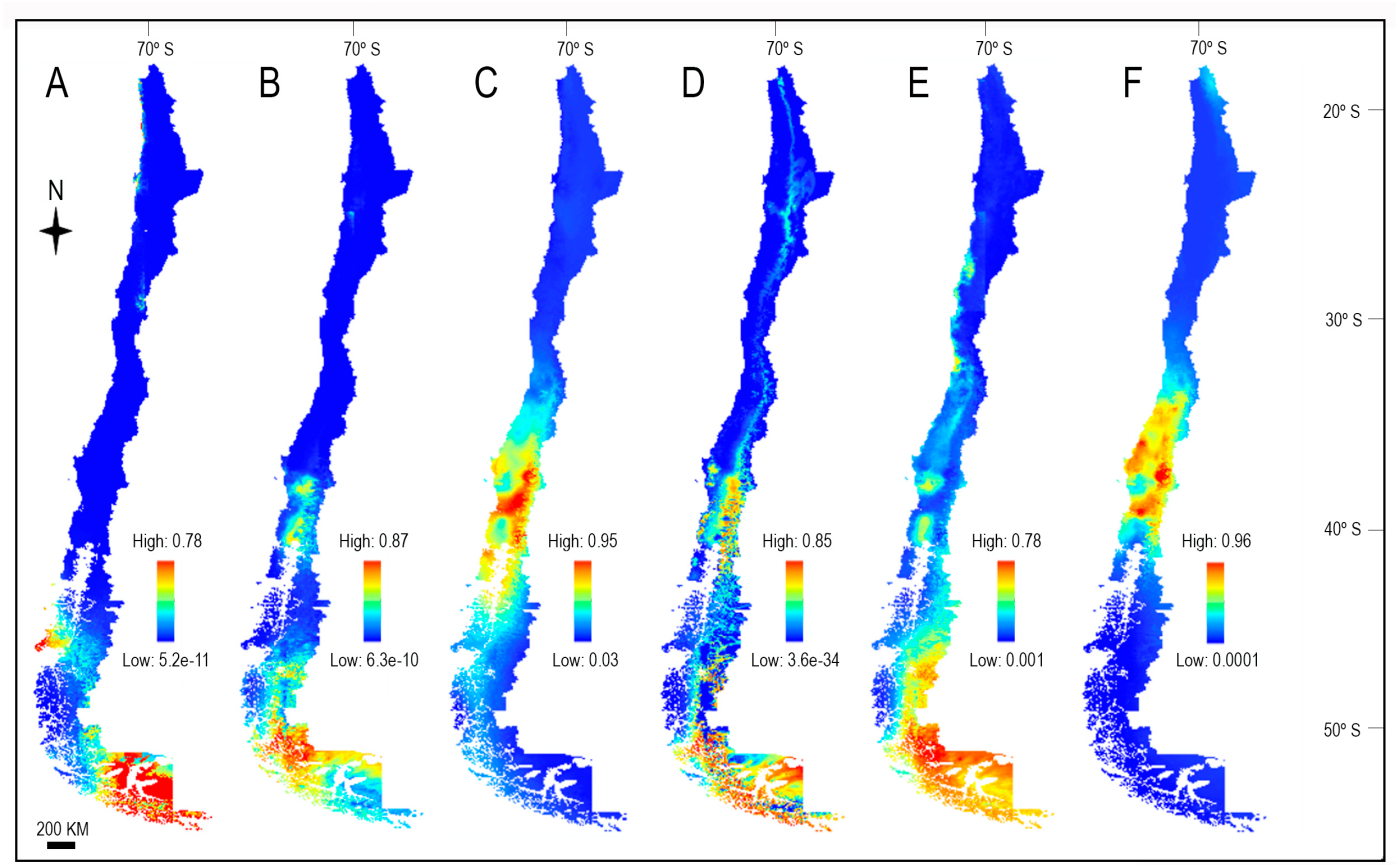
Ecological niche modeling for Chilean *Radiodiscus* spp. generated using MaxEnt. (A) *Radiodiscus australis*, (B) *Radiodiscus coppingeri*, (C) *Radiodiscus flammulatus*, (D) *Radiodiscus magellanicus*, (E) *Radiodiscus riochicoensis*, (F) *Radiodiscus villarricensis*. The maps were created using ArcGIS software (http://www.arcgis.com/index.html). (Maps: GA Collado).

We performed niche modeling in six of the eight species in which the conservation status was evaluated given that *R. coarctatus* and *R. quillajicola* had only one occurrence record and for this reason they were not modeled. Niche modeling results showed that *Radiodiscus* species may occupy potential habitats beyond their current range (Fig. 2), especially *R. riochicoensis* and *R. magellanicus*, whose potential distribution could extend to northern Chile, and *R. flammulatus* to Chiloé Island. Modeling also indicated that different climate and environmental variables influencing the habitat of Chilean *Radiodiscus* species (Table 3). Among all variables, MaxEnt selected the solar radiation as the most influential in the probability of occurrence of *R. australis*, *R. magellanicus* and *R. riochicoensis*, contributing with 29.7%, 43.5% and 65.1% to the model, respectively. For *R. magellanicus*, however, the Minimum temperature of coldest month (Bio6) was also important, accounting for 35.9%. For *R. villarricensis*, the most influential variable was Precipitation of wettest month (Bio13), contributing with 42.7%, while for *R. flammulatus* it was Precipitation of the coldest quarter (Bio19) (87.3%), and for *R. coppingeri* Precipitation of driest quarter (Bio17) (40.7%). The contribution of the other variables to the model in each species is shown in Table 3. The AUC values ranged from 0.79 to 0.97 among species, indicating a good fit of the model (Table 3).

**Table 3:** Ecological niche modeling results with respect to the relative contributions (in percentage) of the climate and environmental variables in species of *Radiodiscus* and model validation.

**Variable/model performance**	***Radiodiscus*** **spp.**					
	***Australis***	***Coppingeri***	***Flammulatus***	***Magellanicus***	***Riochicoensis***	***Villarricencis***
*Climatic*						
bio1						
Bio2	15.6	15.6			3.5	
Bio3		3.6	3.5	6.3	1.7	
Bio4						
Bio5						
Bio6				35.9		
Bio7						
Bio8	3.5					
Bio9						
bio10						
bio11		0.1				
bio12		0.7				
bio13		0.9				42.7
bio14		8.6		4.3		39.3
bio15	14.1	0.9		1.5		
bio16	6.7			6.4		
bio17		40.7				
bio18			9.2			
bio19	19.5	6.5	87.3		23.1	18
*Environmental*						
Elevation	10.9	8.5		2.1	6.6	
Evaporation		1.5				
Solar Radiation	29.7	1.9		43.5	65.1	
Wind		10.5				
*Performance*						
AUC training data	0.97	0.95	0.84	0.92	0.89	0.974
AUC test data	–	0.85	0.92	0.91	0.79	–

**Notes.**

Variables used Bio1 Annual mean temperature bio2 Mean diurnal range (monthly mean: T° max–T° min) bio3 Isothermality bio4 Temperature seasonality bio5 Maximum temperature of warmest month bio6 Minimum temperature of coldest month bio7 Temperature annual range (bio5–bio6) bio8 Mean temperature of wettest quarter bio9 Mean temperature of driest quarter bio10 Mean temperature of the warmest quarter bio11 Mean temperature of coldest quarter bio12 Annual precipitation bio13 Precipitation of wettest month bio14 Precipitation of driest month bio15 Precipitation seasonality bio16 Precipitation of wettest quarter bio17 Precipitation of driest quarter bio18 Precipitation of the warmest quarter bio19 Precipitation of the coldest quarter

## Discussion

The conservation status assessment estimated in the present study shows that *R. australis*, *R. coarctatus* and *R. quillajicola* should be listed as Critically Endangered (CR) by the IUCN Red List and Critically Imperiled (N1) by NatureServe, two equivalent categories that represent the highest threats. Of these three species, the most threatened are *R. coarctatus* and *R. quillajicola* since their distribution is restricted to only one locality (Figs. 1B and 1F). These species, which have few records, were not considered as Data Deficient (DD) due to their endemism, so they were treated as being circumscribed to one or a few localities, as recommended by the IUCN (2014). In addition, this organization also recommends that taxa listed as Data Deficient (DD) should not be treated as if they were not threatened, considering the number of threats species face. Of all species, it can be argued that *R. quillajicola* is the most at-risk species since it inhabits only one locality that does not fall within a protected area. Based on the AOO, *R. coppingeri*, *R. flammulatus*, *R. magellanicus*, *R. villarricensis* and *R. riochicoensis* were listed as Endangered (EN) by the IUCN criteria, which is consistent with the classification of these species as Imperiled (N2) according to NatureServe (except *R. riochicoensis*).

While Mansur (1996a) listed *R. coppingeri* (as *Radiodiscus coppingers* [sic]) as Data Deficient (DD) following IUCN criteria, our national scale evaluation of this species as Least Concern (LC) (EOO) or Endangered (EN) (AOO) is more informative and useful for conservation efforts in Chile. Besides *R. coppingeri*, only three *Radiodiscus* species have been assessed by the IUCN: *Radiodiscus amoenus* Thiele, 1927 and *Radiodiscus compactus* Suter, 1980 from Brazil, listed as Endangered (EN) and Vulnerable (VU), respectively (Mansur, 1996b; Mansur, 1996c), and *Radiodiscus iheringi* Smith, 1881 from Argentina, Brazil, and Uruguay, listed as Data Deficient (DD) (Mansur, 1996d). Under NatureServe, Turgeon et al. (1998) listed *R. millecostatus* as Vulnerable (G3) and *Radiodiscus abietum* (Barker, 1930) as Apparently Secure (G4). No species of *Radiodiscus* has so far been evaluated by the MMA (2021) in Chile.

While the potential distribution models of species are not considered as a parameter to evaluate their conservation status of species through IUCN or NatureServe guidelines, they can be used to determine potential suitable habitats, in addition to knowing the environmental factors that species tolerate (Carvajal-Hernández et al., 2020). *Radiodiscus flammulatus* and *R. villarricensis* inhabit the central-south area of Chile, while *R. australis*, *R. coppingeri*, *R. magellanicus* and *R. riochicoensis* occur in the south-austral zone of the country. Although the distribution patterns of these species appear as highly disjunct, in some cases being separated by hundreds of kilometers, MaxEnt modeling inferred that their distribution areas contain suitable habitats beyond the currently known occurrences and range. However, considering that for *R. australis* and *R. villarricensis* two and three records were used in the modeling, respectively, the derived results should be interpreted as identifying regions that have similar environmental conditions to those present in the occurrence sites of the species (Pearson et al., 2007), *i.e.,* not predicting realized niches. Modeling also indicated that the Chilean *Radiodiscus* species respond to different environmental conditions. These overpredicted areas have been identified as potential habitats that may contain new species, as well as discovering previously unknown ranges (Raxworthy et al., 2003; Menon et al., 2010).

### Recommended conservation measures

To date, there are no studies addressing the genetic diversity of any of the species of *Radiodiscus* in Chile, so it is still unknown whether there is gene flow between populations of more widespread species. Even their basic biology, such as life cycle, reproduction, development, vagility and population sizes, remains to be investigated. Considering these gaps in knowledge, and according to the degree of risk reported here, our first recommendation is to carry out basic studies on the biology of these species, prioritizing the most threatened ones.

Several *in situ* and *ex situ* conservation measures have been proposed in conservation biology (Berkmüller & Savasdiasara, 1981; Bloxam, Tonge & Horton, 1984; Tonge & Bloxam, 1991; CBD, 1992; Pearce-Kelly et al., 1997; Balmford, Leader-Williams & Green, 1995; Mace, Pearce-Kelly & Clarke, 1998; IUCN, 2002; Hadfield, Holland & Olival, 2004; Anonymous, 2005; Kadis, Thanos & Laguna Lumbreras, 2013; Trias-Blasi, Gücel & Özden, 2017). Article 8 of the CBD (1992) proposes 13 *in situ* conservation measures, including the establishment of protected areas, along with its management and protection, to promote the preservation of ecosystems and natural habitats, as well as to prevent the introduction of (or facilitate control or eradication) of exotic species. In accordance with these recommendations, ideally, we propose to enlarge the area of some national parks, create reserves, micro-reserves and environmental interpretive trails to preserve species of *Radiodiscus* in Chile (Berkmüller & Savasdiasara, 1981; Balmford, Leader-Williams & Green, 1995; Laguna et al., 2004). Although the beneficial results of interpretive trails have been questioned (Navrátil, Knotek & Pícha, 2015), the creation of this type of infrastructure remains common in conservation biology as it focuses on the education of the public rather than in all-out preservation of the area and its biota (Berkmüller & Savasdiasara, 1981; Munro, Morrison-Saunders & Hughes, 2008). On the other hand, micro-reserves have proven effective in conservation, where small plots of land of a few hectares are established (Kadis, Thanos & Laguna Lumbreras, 2013; Trias-Blasi, Gücel & Özden, 2017). Micro-reserves are easier to create and can benefit the area and its species, although they need to be established as a network of reserves to succeed (Lumbreras, 2001). Their success for plant species (Laguna et al., 2016) might be a good sign for micromollusks with supposed reduced mobility.

Article 9 of the CBD (1992) also proposes five *ex situ* measures that may be implemented for species conservation. Some *ex situ* conservation efforts have been developed through captive breeding programs to preserve tree snails of the families Achatinellidae and Partulidae (Bloxam, Tonge & Horton, 1984; Tonge & Bloxam, 1991; Pearce-Kelly et al., 1997; Mace, Pearce-Kelly & Clarke, 1998; Hadfield, Holland & Olival, 2004), endemic to Hawaii and islands of the south-western tropical Pacific Ocean, respectively, which face extinction. However, up to our knowledge there are no reports of successful breeding of minute Charopidae snails such as *Radiodiscus* spp., so the feasibility of this approach is uncertain.

Some *in situ* and *ex situ* conservation measures are proposed below for Chilean *Radiodiscus* species. The benefits and drawbacks of each approach must be assessed and weighted against costs and immediate and potential benefits. Realistically, not all of these measures will be feasible in all concerned areas in Chile and, as such, they should be assessed on a case-by-case basis alongside policy makers.

*Radiodiscus australis* (Fig. 1A) presents only two occurrences in Chile, none of them within a protected area. It is exposed to a high level of nature and sports tourism, and forestry activities (exploitation of native forests for wood, firewood and paper and livestock disturbance) (Magallanes, 2012; Arredondo et al., 2019). Furthermore, the area is inhabited by beavers (*Castor canadensis* Kuhl, 1820), an invasive species that drastically modifies the habitat (Graells, Corcoran & Aravena, 2015), although its net impact on the terrestrial snail fauna remains unstudied. Moreover, the Brunswik Peninsula has coal reservoirs (Magallanes, 2012), the exploitation of which could become a threat to the species in the near future. Today, *ex situ* conservation actions such as captive breeding should be trialed as a priority to preserve the species. We also recommend the implementation of *in situ* conservation actions such as environmental interpretive trails and beaver eradication where *R. australis* populations occur. The implementation of micro-reserves would also be an appropriate conservation measure.

*Radiodiscus coarctatus* (Fig. 1B) faces threats associated to tsunamis, tourism, artisanal fishing and soil erosion due to heavy rainfalls and strong winds (DAPMA, 2009), although adverse weather conditions in turn reduce tourism (CONAF, 2021). Although the species was described from Caleta Nantuel, Vidal Gormaz Island, within the Kawésqar National Park (Fig. 1B), we recommend the implementation of an environmental interpretation trail and implement *ex situ* conservation programs as mentioned above.

*Radiodiscus coppingeri* (Fig. 1C) faces threats in many fronts, such as tourism, soil erosion due to rainfall (storms), activities associated to artisanal fishing (DAPMA, 2009), solid contamination caused by the influx of tourists (El Heraldo Austral, 2019), illegal logging of native forest (Magallanes, 2012; Mardones, 2020), risk of forest fires (CONAF, 2020), and change in land use due to livestock (Arredondo et al., 2019), as well as the presence of beavers in austral Patagonia (Graells, Corcoran & Aravena, 2015; GEF, 2019). In addition, several populations are close to urban areas, volcanoes, hydrocarbon mines (SONAMI, 2021) and coal reserves (Martinic, 2004; Magallanes, 2012). Even though *R. coppingeri* is the species with the highest number of occurrences, the conservation category resulting from this study was Endangered (EN) according to IUCN criteria (AOO) and Imperiled (N2) under NatureServe criteria (Table 2). We recommend enlarging the Bernardo O’Higgins National Park since there are five occurrences located on the eastern margin of the Serrano River. We also recommend the creation of environmental interpretive trails in different localities where this species occurs, as well as beaver eradication.

*Radiodiscus flammulatus* (Fig. 1D) is threatened by tourism and recreational activity, such as camping, that causes solid contamination (El Heraldo Austral, 2019; Robles, 2021). On the other hand, the Laguna del Laja National Park borders on infrastructure associated with the El Toro hydroelectric plant, causing droughts in the area (Arnaboldi, 2016; Díaz, Jaque & Ojeda, 2018). Because *R. flammulatus* is Endangered (EN) or Imperiled (N2) according to our assessment, we suggest enlarging the Alerce Andino National Park since there is one occurrence approximately 0.1 km outside the park towards the V-657 highway. Similarly, we also suggest enlarging the Bernardo O’Higgins National Park (as for *R. coppingeri* above) because there is another occurrence located approximately 1.6 km from the park towards Lake Brush. We also recommend the creation of environmental interpretive trails since some of the threats mentioned above affect even the interior of protected areas.

*Radiodiscus magellanicus* (Fig. 1E) faces various threats such as monoculture forestry and exploitation of native trees (Magallanes, 2012), livestock activities for consumption and export (Arredondo et al., 2019), heavy rainfall (storms), high tourist activity and presence of the invasive beaver (Graells, Corcoran & Aravena, 2015; GEF, 2019). In addition, several populations of the species are located near volcanoes, highways/roads, urban areas and hydrocarbon mines. We recommend the creation of environmental interpretive trails in addition to enlarging the Bernardo O’Higgins National Park (as for *R. coppingeri* and *R. flammulatus* above), since there is one occurrence approximately 1 km from the park towards Lake Brush. We also recommend beaver eradication.

*Radiodiscus quillajicola* (Fig. 1F) inhabits a single locality near a busy highway with potential for landslides (Cooperativa, 2016; Arenas, 2017). In addition, this locality is not a protected area and is close to the Laguna del Maule volcanic complex, which currently has a yellow alert level (SERNAGEOMIN, 2021). Thus, the severity of the threats to this species is high. The trialing and implementation of *ex situ* conservation actions such as captive breeding should be considered a priority to preserve the species. We also recommend the creation of a micro-reserve to protect both the species and its habitat, in addition to implementing an environmental interpretation trail and surveying the neighboring area for eventual occurrences of the species.

*Radiodiscus riochicoensis* (Fig. 1G) faces serious threats such as the presence of the invasive beaver (Graells, Corcoran & Aravena, 2015; GEF, 2019), forestry activities for the exploitation of native forests (Magallanes, 2012), livestock activity focused on export (Arredondo et al., 2019), activities associated with artisanal fishing (DAPMA, 2009), pollution (El Heraldo Austral, 2019), risk of forest fires (Vidal et al., 2015), heavy rainfall (storms), and a high degree of tourism and recreational activities. In addition, some populations are located close to volcanoes, highways, urban areas, coal reservoirs (Magallanes, 2012) and hydrocarbon mines (SONAMI, 2021). We recommend the creation of environmental interpretive trails and micro-reserves for the protection of the species, as well as beaver eradication.

The three localities where *R. villarricensis* inhabits (Fig. 1H) face a large amount of tourism and recreational activities (Subsecretaría de Turismo, 2017; Morales Flores, 2019) and the risk of forest fires (Villavicencio, 2015; Morales Flores, 2019; Enríquez, 2021). In addition, two of the three occurrences are close to urban areas and one to volcanoes. We recommend the trialing and implementation of *ex situ* conservation programs, the creation of environmental interpretive trails, and the creation of a micro-reserve in Fundo El Manzano, Bío-Bío Region. The feasibility of creating a national reserve in Cerro Caracol, Concepción city, should also be taken into consideration.

Identifying threatened species is one of the main tasks of conservation biology, following with an assessment of the threats and the development of strategies to remediate the situation (Purvis et al., 2000; Barbini et al., 2020). In the present study, we proposed *in situ* and *ex situ* conservation measures to preserve eight species of *Radiodiscus* in Chile, most of which are highly threatened. However, although these types of actions are not exempt from difficulties, such as the cost of the land, its management, territorial custody, associated facilities, staff and the type of feed and appropriate abiotic environmental conditions in the case of artificial rearing systems (Hadfield, Holland & Olival, 2004; O’Rorke et al., 2016; Kadis, Thanos & Laguna Lumbreras, 2013; Laguna et al., 2004; Carranza et al., 2020), they have been rather promising and therefore applicable to preserve species, including small snails.

## Conclusions

This study assesses for the first time the conservation status of *Radiodiscus* species using IUCN and NatureServe standards in parallel. Both systems categorized the eight Chilean species evaluated in categories of serious threat. The distribution of each of the species was also updated and the potential threats affecting them were identified. It is strongly recommended to carry out studies of the basic biology of the species and to implement conservation measures in the short term, especially for those that present a high risk of extinction.

##  Supplemental Information

10.7717/peerj.14027/supp-1 Table S1 Details and results of georeferencing of Chilean *Radiodiscus* species Empty cells mean no information or not applicable.

10.7717/peerj.14027/supp-2 Table S2 List of threats identified for species of *Radiodiscus* in Chile

## Additional Information and Declarations

### Data Availability

The following information was supplied regarding data availability: The raw data is available in the Supplemental Files.
