# Supplementary material for: Distribution, conservation status and proposed measures for preservation of Radiodiscus microgastropods in Chile
Source: PeerJ. 2023 Jan 10;11:e14027. doi: 10.7717/peerj.14027 (PMC9838206; doi:10.7717/peerj.14027)
Supplement: Table S2 [file peerj-11-14027-s002.docx]

| **Supplementary Material** |  |  |  |  |
| --- | --- | --- | --- | --- |
| **Article:** Distribution, conservation status and proposed measures for preservation of *Radiodiscus* microgastropods in Chile. | | | | |
| **Authors:** Gonzalo A. Collado, Rodrigo B. Salvador, Marcela A. Vidal, Fernanda Parra, Vannia Delgado and Cristian Torres-Díaz. | | | | |

**Table S3.** List of threats identified for species of *Radiodiscus* in Chile.

| **Species** | **Energy production and mining** | **Urbanization** | **Tourism** | | **Agriculture** | | **Fires** | **Geological events** | **Invasive species** | | **Roads** | **Severe weather** | **Biological resource**  **use** | **Garbage and solid waste** |
| --- | --- | --- | --- | --- | --- | --- | --- | --- | --- | --- | --- | --- | --- | --- |
| *Radiodiscus*  *australis* | X | - | X | X | | - | | - | X | - | | - | X | - |
| *Radiodiscus coarctatus* | - | - | X | - | | - | | - | - | - | | X | X | - |
| *Radiodiscus coppingeri* | X | X | X | X | | X | | X | X | X | | X | X | X |
| *Radiodiscus flammulatus* | X | X | X | - | | - | | X | - | X | | X | - | X |
| *Radiodiscus magellanicus* | X | X | X | X | | - | | X | X | X | | X | X | - |
| *Radiodiscus quillajicola* | - | - | - | - | | - | | X | - | X | | - | - | - |
| *Radiodiscus riochicoensis* | X | X | X | X | | X | | X | X | X | | X | X | X |
| *Radiodiscus villarricensis* | - | X | X | - | | X | | X | - | - | | - | - | - |
